# Indole-3-Carbinol Stabilizes p53 to Induce miR-34a, Which Targets LDHA to Block Aerobic Glycolysis in Liver Cancer Cells

**DOI:** 10.3390/ph15101257

**Published:** 2022-10-13

**Authors:** Yuehua Qi, Chunjing Zhang, Di Wu, Yue Zhang, Yunfeng Zhao, Wenjuan Li

**Affiliations:** 1 College of Basic Medicine, Hebei University, Baoding 071000, China; 2 Key Laboratory of Pathogenesis Mechanism and Control of Inflammatory-Autoimmune Diseases in Hebei Province, Hebei University, Baoding 071000, China; 3 Department of Biochemistry and Molecular Biology, Qiqihar Medical University, Qiqihar 161006, China; 4 Department of Pharmacology, Toxicology & Neuroscience, LSU Health Sciences Center in Shreveport, Shreveport, LA 71103, USA

**Keywords:** aerobic glycolysis, LDHA, I3C, miRNA, liver cancer

## Abstract

Certain cancer cells prefer aerobic glycolysis rather than oxidative phosphorylation for energy supply. Lactate dehydrogenase A (LDHA) catalyzes the reduction of pyruvate to lactate and regains NAD+ so that glycolysis is continued. As a pivotal enzyme to promote smooth glycolysis, LDHA plays an important role in carcinogenesis. Indole-3-carbinol (I3C) has displayed antitumor activity, but the exact mechanism remains to be identified. In this study, we treated liver cancer cells with I3C, performed colony formation and cell migration, measured the expression of glycolysis-related proteins, and predicted and validated LDHA-targeting miRNA from the databases. In addition, the mRNA and protein levels of p53, glycolysis-related genes and miRNAs that regulate glycolysis were detected after I3C and siRNA-p53 treatment alone or in combination. Next, the expression and colocalization of p53 and MDM2 in liver cancer cells were evaluated after I3C treatment, and the effect of I3C on p53 protein stability was examined. The results showed that I3C inhibited cell proliferation, migration, and the expression levels of glycolysis-related gene LDHAs. MiR-34a was predicted to target LDHA, and I3C downregulated its expression. Furthermore, the combined I3C and siRNA-p53 treatment demonstrated that I3C regulated the expression of LDHA via miR-34a in a p53-dependent manner. Finally, I3C inhibited MDM2 expression and its colocalization with p53 and stabilized p53 expression. In summary, I3C inhibited the degradation of p53 by MDM2 in liver cancer cells; stable p53 induced miR-34a, which targeted LDHA, a key enzyme for aerobic glycolysis, suggesting cancer metabolism is an important target for I3C in liver cancer cells.

## 1. Introduction

Metabolic reprogramming has attracted widespread attention because it runs through the courses of carcinogenesis [1]. Even under normoxia, certain cancer cells tend to obtain energy via glycolysis, known as the “Warburg effect” or “Aerobic glycolysis” [2]. Aerobic glycolysis inhibitors that target deregulated cellular energetics have been studied for cancer treatment [1]. For instance, lactate dehydrogenase (LDH) has been proposed as a possible target for cancer diagnosis, therapy, and prevention [3]. LDH catalyzes a reversible reaction between pyruvate and lactate, whereas LDH isoform A (LDHA) tends to metabolize pyruvate to lactate, and LDH isoform B (LDHB) catalyzes the reverse reaction [3]. Studies have found that LDHA overexpression is inversely correlated with survival in solid tumors and also associated with drug resistance [4]; downregulated LDHA promotes oxidative phosphorylation (OXPHOS), which is reversed by electron transport chain inhibitors [5]. The regulation of LDHA expression involves multiple levels [6]; one of these is post-transcriptional processing, which affects mRNA stability. MicroRNAs (miRNAs) are short, endogenously initiated, noncoding RNAs that repress gene translation by inducing mRNA degradation [7]. Studies have shown that compounds inhibit tumor proliferation, invasion, and migration through miR-34a in gastric cancer cells [8]; in breast cancer cells, glycolysis and cell proliferation can be directly inhibited by miR-34a [9].

p53, coded by the gene p53, is often referred to as the “guardian of the genome” as it protects the integrity of DNA in tissue cells and regulates cell cycle arrest, DNA damage repair, and apoptosis [10]. In addition, p53 regulates the expression of noncoding RNA in addition to protein-coding genes, and microRNAs (miRNAs) are one of them [11]. Furthermore, the stability and expression of p53 are also subject to various regulations. p53 is modified by post-translational modifications such as acetylation, phosphorylation, and ubiquitination, etc. [12]. The ubiquitination of p53 is predominantly mediated by murine double minute 2 (MDM2), an E3 ubiquitin ligase involved in the proteasome pathway [13]. Regulating metabolism is an important function of p53. p53 directly inhibits the transcription of glucose transporter 1 (GLUT1) and GLUT4, thereby reducing glucose uptake [14]. Glycolysis could be inhibited by p53-downregulated hexokinase 2 (HK2) in prostate cancer cells [15]; p53 induces the expression of E3 ubiquitin ligase Parkin to promote hypoxia inducible factor-1α (HIF-1α) degradation through ubiquitination and subsequently inhibit the expression of glycolysis enzymes LDHA and GLUT1 in breast cancer MCF7 cells [16].

As a compound found in cruciferous vegetables, Indole-3-carbinol (I3C) is derived from the breakdown of glucobrassicin [17]. Its safety in normal cells has been proven [18], and it has displayed plenty of anticancer activities [19]. For example, I3C inhibits the proliferation of breast cancer cells through the NF–κB pathway [20]; I3C regulates the expression of apoptosis-related proteins and induces G1 phase cell-cycle arrest in lung cancer cells [21]; in liver cancer cells, I3C induces DNA damage and increases the permeability of mitochondria and the release cytochrome C from mitochondria into the cytoplasm [22]. In addition, I3C exerts a potential role in the energetic metabolism of cells. In HeLa cells, I3C reduces intracellular glucose and lactate concentrations and inhibits glucose metabolism [23]. I3C could activate p53, thereby directly or indirectly regulating the expression of glycolysis-related proteins such as GLUT, LDHA, and PKM2 [24]. More importantly, I3C is a natural inhibitor of E3 ubiquitin ligase WWP1, which in turn triggers the ubiquitination of PTEN and regulates PKM2 during aerobic glycolysis [25].

The latest global cancer (GLOBOCAN) statistics has shown that primary liver cancer ranks sixth in incidence and third in mortality [26]. In China, the incidence and mortality of liver cancer are fifth and second, respectively [27]. Liver is the main metabolic organ, and studies have suggested that combinational therapies targeting autophagy and aerobic glycolysis may be effective in the treatment of liver cancer [28]. Other studies have also demonstrated that downregulation of Forkhead box protein K1 (FOXK1) regulates aerobic glycolysis, thereby inhibiting liver cancer cell proliferation [29]. The metabolic reprogramming has been confirmed throughout the development of cancer [30]. It is worth exploring the exact molecular mechanism of I3C in this process and investigating the therapeutic potential of I3C in human liver cancers, which may provide more effective theoretical and experimental bases for liver cancer diagnosis and treatment.

## 2. Results

### 2.1. I3C Suppresses Liver Cancer Cells Proliferation and Migration and Inhibits Aerobic Glycolysis

**Figure 1:**
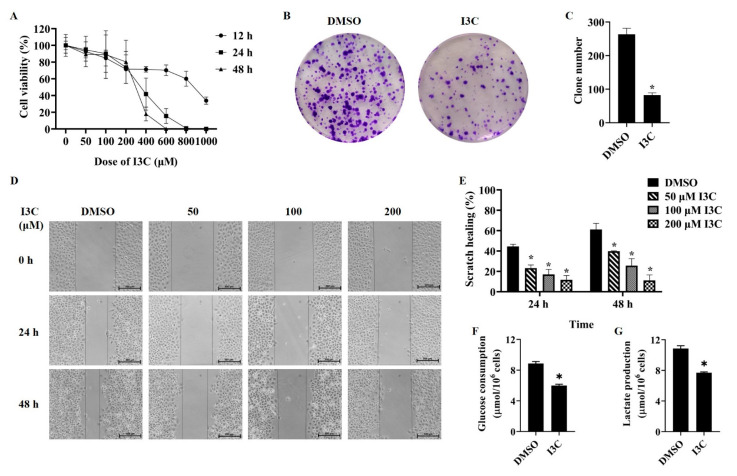
The effects of I3C treatment on cell proliferation, colony formation, migration, and glycolysis in HepG2 cells. (**A**) The CCK8 assay in HepG2 cells, which were treated with different time and dose from I3C; (**B**) Colony formation assay after vehicle (DMSO) and I3C treatments. (×40); (**C**) The number of colonies; (**D**) Wound scratch assay after vehicle (DMSO) and I3C treatments. (×10); (**E**) The wounded area, shown as an arbitrary unit. The effects of I3C treatment on the glucose consumption (**F**) and the lactate production (**G**) in HepG2 cells. * *p* < 0.001 vs. DMSO.

To validate whether I3C has an effect on liver cancer cell growth, the CCK8 assay was performed on liver cancer HepG2 cells, and a dose- and time-dependent suppression of cell proliferation were observed (Figure 1A). Based on the IC50 (949 μM for 12 h, 282 μM for 24 h, 235 μM for 48 h), 200 μM I3C and 12 h treatment were chosen for subsequent studies. Unlimited division of cancer cells is a key feature, which was measured using a clonogenic assay in HepG2 cells treated with I3C for 12 h. The data showed that, compared with the control group, I3C significantly inhibited the colony formation of HepG2 cells (Figure 1B,C). To detect cell migration ability, another feature of cancer cells, scratch wound assay was utilized, and, as shown in Figure 1D,E, I3C treatment retarded the healing of the scratch area in a time- and dose-dependent manner in HepG2 cells. Next, whether I3C regulates glycolysis in liver cancer cells was studied. The results demonstrated that I3C treatment significantly inhibited lactate production and glucose consumption (Figure 1F,G), suggesting that I3C is effective to impede the glycolytic phenotype of liver cancer cells.

**Figure 2:**
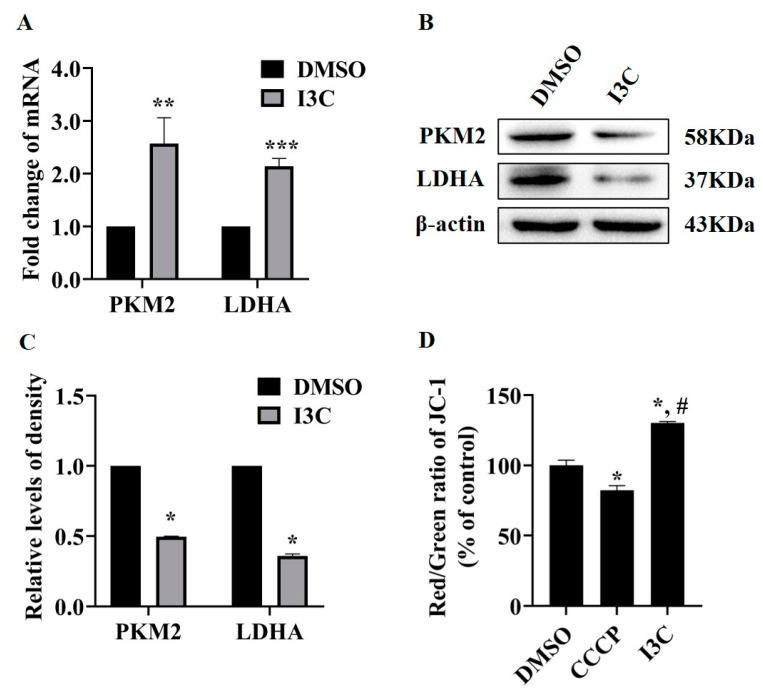
The effects of I3C treatment on the expression of glycolysis-related genes and mitochondrial function in HepG2 cells. (**A**) Measurement of the mRNA levels (** *p* < 0.01 vs. DMSO, *** *p* < 0.001 vs. DMSO) and (**B**) protein levels of glycolysis-related genes after DMSO (vehicle) and I3C (200 µM for 12 h) treatments; (**C**) Densitometric analysis for blots shown in (**B**). * *p* < 0.05 vs. DMSO; (**D**) Effects of I3C on MMP. The mitochondrial uncoupler CCCP was used as a control. * *p* < 0.05 vs. DMSO, # *p* < 0.05 vs. CCCP.

Glycolysis includes multiple chemical reactions, and some enzymes are involved in this process [31]. To address whether I3C directly affects glycolysis, the mRNA and protein expression levels of glycolysis-related enzymes were measured using qPCR and Western blot, respectively. The results showed that I3C treatment increased the mRNA levels of PKM2 and LDHA, both important glycolysis-related genes (Figure 2A). However, the proteins level of PKM2 and LDHA were decreased after I3C treatment (Figure 2B,C). Meanwhile, we detected the change in mitochondrial membrane potential (MMP) as a marker for mitochondrial function [32]. As shown in Figure 2D, the levels of MMP in the I3C treatment group were significantly higher than that in the control group. These results suggest that I3C affects the expression of glycolysis-related proteins, not at the RNA level, and these changes negatively correlate with MMP. To study how I3C regulates the expression of glycolysis-related genes at the post-transcriptional level, in the following experiments we focused on LDHA because it plays a key role in the regeneration of nicotinamide adenine dinucleotide (NAD+), which is required to maintain glycolysis [33].

### 2.2. I3C Regulates LDHA Expression through miR-34a in a p53-Dependent Manner

**Figure 3:**
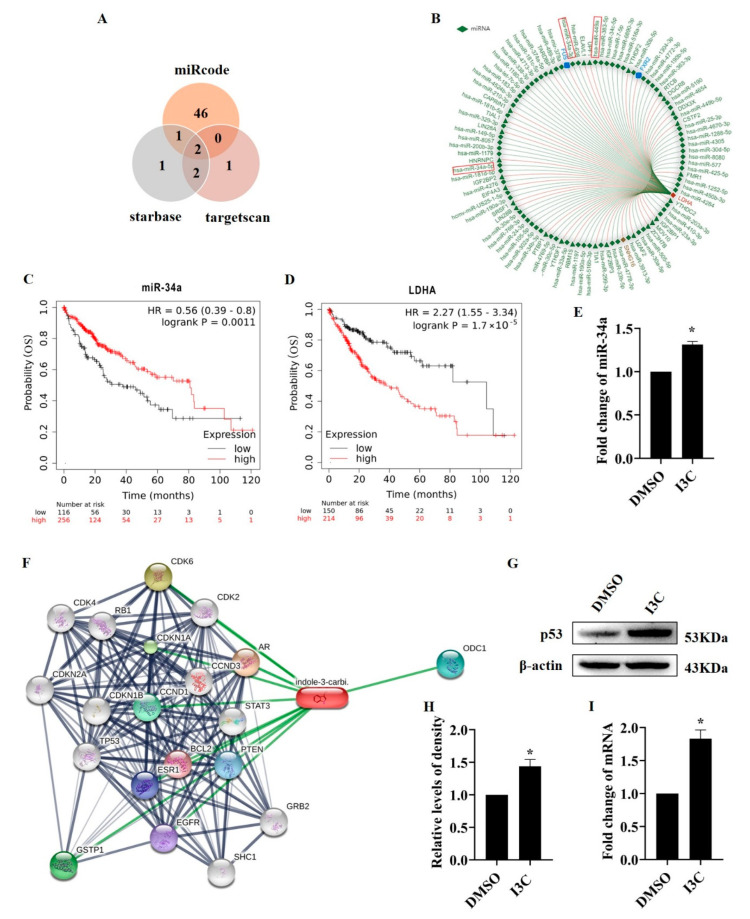
Studies on the molecular mechanism of how I3C treatment inhibited LDHA expression. (**A**) LDHA-targeted miRNAs were predicted using three databases; (**B**) Correlation of miR-34a with LDHA expression in hepatocellular carcinoma tissues. Association between the expression levels of miR-34a (**C**) and LDHA (**D**) and survival in patients with liver cancer; (**E**) The effects of I3C treatment on the expression of miR-34a in HepG2 cells. * *p* < 0.05 vs. DMSO; (**F**) Prediction of the protein–chemical interaction. (**G**) The effect of I3C treatment on p53 protein expression; (**H**) Densitometric analysis for blots shown in (**G**). * *p* < 0.05 vs. DMSO; (**I**) Measurement of the mRNA levels of p53 after treatment with DMSO (vehicle) and I3C. * *p* < 0.05 vs. DMSO.

It is known that miRNAs are key molecules in the post-transcriptional regulation of LDHA [9]. We found that both miR-34a and miR-449a can target LDHA using three online tools (miRcode, starbase, and targetscan) (Figure 3A). RNAlocate was used to validate our prediction (Figure 3B). The relationship between the expression levels of miR-34a and LDHA and liver cancer patient survival rate was examined using the Kaplan–Meier Plotter tool. The results showed that patients with low miR-34a expression had lower overall survival and poor prognosis (Figure 3C), whereas patients with high LDHA expression had worse survival (Figure 3D). Next, we studied the effect of I3C treatment on miR-34a. The results showed that I3C treatment increased the miR-34a level (Figure 3E).

To figure out how miRNA is regulated by I3C treatment, we utilized the STITCH tool to predict the regulators being involved. The result showed that p53 can be the molecule that plays a regulatory role (Figure 3F), which is consistent with several other studies [34]. Indeed, our results showed that I3C treatment increased the protein (Figure 3G,H) and mRNA (Figure 3I) levels of p53.

**Figure 4:**
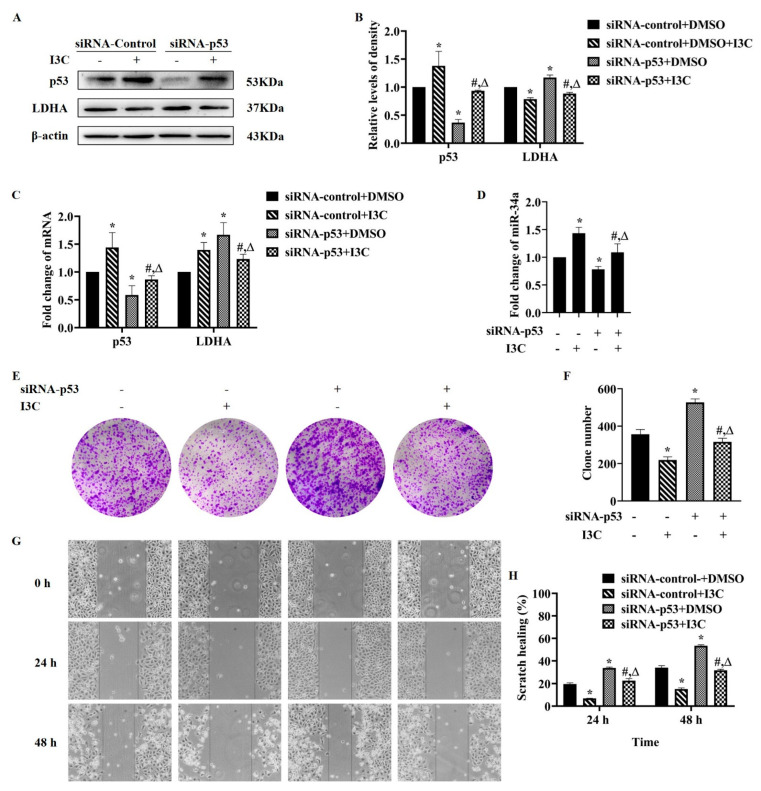
I3C regulated LDHA expression through miR−34a in a p53-dependent manner. HepG2 cells were treated with I3C and siRNAs alone or in combination. (**A**) The effects on the protein expression of p53 and LDHA; (**B**) Densitometric analysis for blots shown in (**A**). * *p* < 0.05 vs. siRNA−control+ DMSO, # *p* < 0.05 vs. siRNA−control +I3C; (**C**) Measurement of the mRNA levels of p53 and LDHA; (**D**) The effect on the expression of miR-34a; (**E**) Colony formation assay. (×40); (**F**) Statistical analysis using the colony numbers; (**G**) Wound scratch assay. (×10); (**H**) Wounded area, shown as an arbitrary unit. * *p* < 0.05 vs. siRNA−control+ DMSO, # *p* < 0.05 vs. siRNA−control +I3C, Δ *p* < 0.05 vs. siRNA−p53+ DMSO.

Next, how I3C regulates LDHA-targeted miRNA via p53 was studied in p53 knockdown HepG2 cells. As shown in Figure 4A,B, p53 knockdown upregulated LDHA expression, which was reversed after I3C treatment. As for the mRNA levels of p53 and LDHA, the former was in line with the changes in protein levels, whereas the latter was different, as expected (Figure 4C). The results in Figure 4D demonstrated that I3C treatment reversed the downregulation of miR-34a caused by knockdown of p53. We then performed colony formation and wound scratch assays for phenotype evaluation. The results showed that, compared with the control group, siRNA-p53 promoted colony formation (Figure 4E,F) and migration (Figure 4G,H) of HepG2 cells, whereas I3C treatment attenuated these promotional effects. These results demonstrate that I3C inhibits LDHA expression via the p53/miRNA-34a axis.

**Figure 5:**
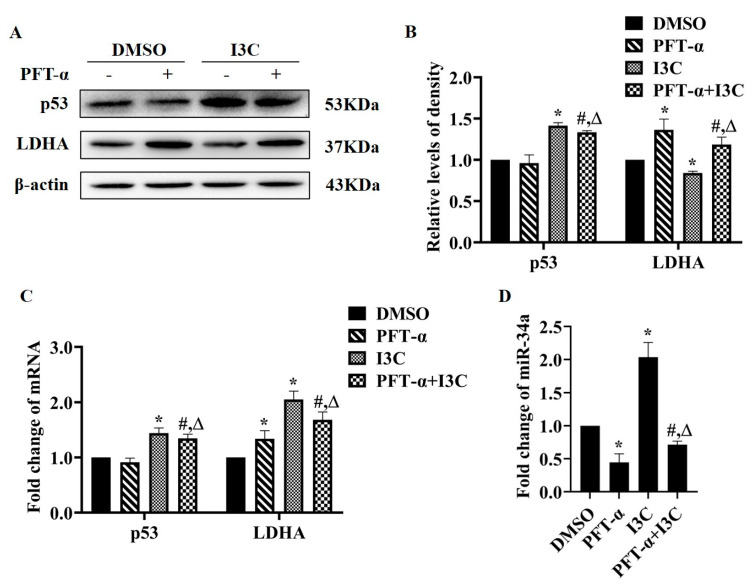
The effects of I3C and p53 inhibitor (PFT-α) on the expression levels of miR-34a and LDHA. (**A**) The effect on the protein expression of p53 and LDHA; (**B**) Densitometric analysis for blots shown in (**A**); (**C**) Measurement of the mRNA levels of p53 and LDHA; (**D**) The effect on the expression of miR-34a. * *p* < 0.05 vs. DMSO, # *p* < 0.05 vs. PFT-α, Δ *p* < 0.05 vs. I3C.

PFT-α, as an inhibitor of p53 transcription activity, was used to further confirm the relationship between p53, miRNA, and LDHA. Based on CCK8 analysis (Figure S1), we chose 20 µM of PFT-α for the 12 h treatment for the following experiments. Firstly, PFT-α treatment enhanced the protein and mRNA levels of LDHA with no significant effects on that of p53; I3C treatment attenuated the effect of PFT-α on the protein levels (Figure 5A,B), not mRNA levels, of LDHA (Figure 5C). PFT-α treatment suppressed the expression of miR-34a, which was inhibited by I3C (Figure 5D). These data further suggest that I3C regulates the expression of LDHA via the p53/miR-34a axis.

### 2.3. I3C Treatment Downregulates MDM2 and Stabilizes p53

**Figure 6:**
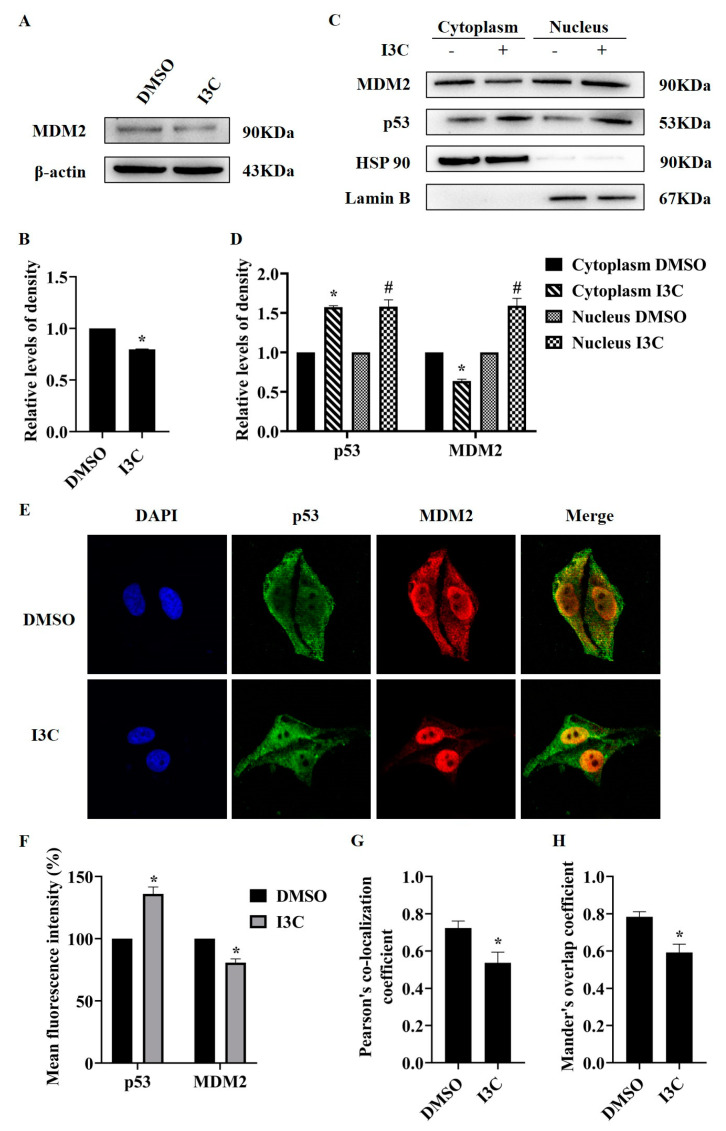
The effects of I3C treatment on MDM2 and p53. (**A**) The effect of I3C treatment on MDM2 expression; (**B**) Densitometric analysis for blots shown in (**A**). * *p* < 0.05 vs. DMSO; (**C**) Effect of I3C treatment on the protein levels of MDM2 and p53 in the nucleus and cytoplasm. Lamin B as a loading control for nuclear fraction and HSP90 for cytoplasmic fraction; (**D**) Densitometric analysis for blots shown in (**C**). * *p* < 0.05 vs. Cytoplasm DMSO, # *p* < 0.05 vs. Nucleus DMSO; (**E**) The subcellular localization of MDM2 and p53 with immunofluorescence staining (×40); (**F**) Analysis of the mean intensity of immunofluorescence; (**G**) Pearson’s correlation coefficiency analysis; (**H**) Manders’ Colocalization Coefficiency analysis. * *p* < 0.05 vs. DMSO.

**Figure 7:**
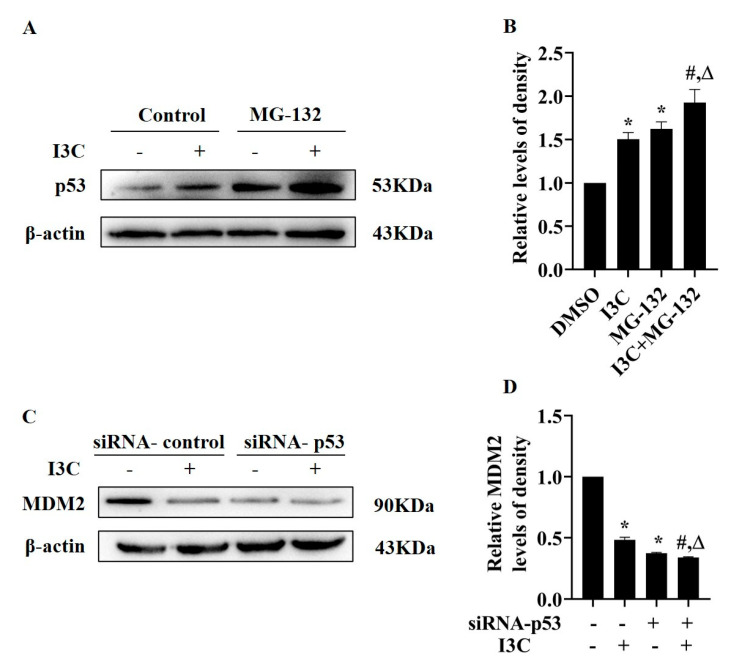
The effect of I3C on the stabilization of p53 by MDM2. (**A**) The effect of combined I3C and MG-132 on the expression of p53; (**B**) Densitometric analysis for blots shown in (**A**). * *p* < 0.05 vs. DMSO, # *p* < 0.05 vs. I3C, Δ *p* < 0.05 vs. MG-132; (**C**) The effect of I3C on MDM2 expression in siRNA-p53 transfection cells; (**D**) Densitometric analysis for blots shown in (**C**). * *p* < 0.05 vs. siRNA-control+DMSO, # *p* < 0.05 vs. siRNA-control+I3C, Δ *p* < 0.05 vs. siRNA-p53+DMSO.

Since the ubiquitination by MDM2 is a primary passion in the post-translational regulation of p53, we investigated whether I3C plays a role here. We first detected MDM2 expression, and I3C treatment reduced the protein levels of MDM2 (Figure 6A,B). To further assess the effect of I3C on the interaction of p53 and MDM2, we extracted the nuclear and cytoplasmic fractions of HepG2 cells after I3C treatment. As shown in Figure 7C,D, the protein levels of MDM2 were increased in the nucleus but decreased in the cytoplasm, while the protein levels of p53 were increased in both nucleus and cytoplasm after I3C treatment. Meanwhile, the results from the immunofluorescence assay showed the mean fluorescence intensity of p53 was increased, whereas the mean fluorescence intensity of MDM2 was decreased after I3C treatment (Figure 6E,F); furthermore, the colocalization coefficiency of p53 and MDM2 was reduced after I3C treatment (Figure 6G,H). These findings suggest that: (1) I3C upregulates p53 expression and promotes its transcriptional activity in the nucleus; (2) I3C might inhibit the activity of MDM and thereby stabilize p53.

Finally, we studied the effect of I3C treatment on the degradation of p53. HepG2 cells were treated with the proteasome inhibitor MG-132 and I3C alone or in combination; the results showed increased p53 expression when MG-132 was combined with I3C (Figure 7A,B). Next, we measured the protein levels of MDM2 in siRNA-p53 transfected HepG2 cells and observed that knockdown of p53 inhibited the expression levels of MDM2 (Figure 7C,D), suggesting that there is feedback regulation between p53 and MDM2, which is consistent with other studies [35]. Altogether, these results indicate that I3C stabilizes p53 expression by inhibiting its degradation to ensure its transcriptional activity.

## 3. Discussion

Due to the rapid growth and division of cancer cells, the metabolic mode has been changed to gain energy through glycolysis irrespective of oxygen availability, which is known as “metabolic reprogramming” [2]. So far, scientists have proposed 14 key hallmarks of cancer, including limitless replicative potential [36], deregulating cellular energetics [1], senescent cells [37], etc., which all well explain the mechanism of carcinogenesis and therapeutic response. Plants as a source of medicines have been used clinically in the treatment of various diseases including cancer. I3C is a natural agent widely present in cruciferous vegetables, and its safety on normal cells has been confirmed [18]. I3C has shown great potential in cancer prevention and chemotherapy [19]. Studies have pointed out that I3C inhibits cell proliferation, induces apoptosis, and promotes cell cycle arrest to inhibit cancer development through various pathways [21,38]. Based on our results, metabolic reprogramming may serve as a new mechanism for I3C’s anticancer activity.

Through a series of experiments, our data have demonstrated that I3C inhibits the glycolytic phenotype as well as the clonal proliferation, migration of liver cancer cells, glucose consumption, and lactate release (Figure 1). These data suggest that I3C may function as an aerobic glycolysis inhibitor, which can deregulate cellular energetics to suppress cancer cell growth.

Glycolysis is a multistep process requiring the activities of a number of enzymes including LDHA. LDHA contributes to the regeneration of NAD+ during the reduction of pyruvate to lactate, which is critical for ongoing glycolysis; meanwhile, elevated lactate level is associated with tumor cell proliferation, angiogenesis, and invasion [39,40]. Therefore, LDHA may serve as a potential target for cancer diagnosis and treatment. Our results show that I3C inhibits the protein levels of LDHA, but not the mRNA levels (Figure 2), suggesting I3C regulates LDHA at the post-transcriptional level. Through database prediction and verification, we identified miR-34 as the miRNA involved in the regulation of LDHA, and we experimentally confirmed the effect of I3C on miR-34a expression (Figure 3A–E).

p53 regulates the expression and processing of miRNAs; the latter are involved in carcinogenesis through mediating multiple processes such as cell migration, metabolism, and cell survival [11]. Based on our results, we propose that I3C may regulate miR-34 through p53, as I3C increased the protein levels of p53 (Figure 3F–H). Next, we suppressed the expression of p53 in HepG2 cells by two approaches: siRNA-p53 and a p53 transcriptional activity inhibitor. siRNA-p53 increased the protein, but not the mRNA levels of LDHA (Figure 4). Similar results were obtained using PFT-α, an inhibitor of transcriptional activity of p53 (Figure 5). All these data suggest that the inhibition of LDHA expression by I3C is mediated through p53-upregulated miR-34a.

Subsequently, how I3C regulates p53 was studied. The stability and intracellular localization of p53 have been focal in the molecular mechanisms of cancer inhibition [41]. Building on the previous results, we proposed that I3C may enhance the transcriptional activity of p53. MDM2 binds to the carboxyl-terminal of p53, which induces its nuclear export [10]. We found that the protein expression of MDM2 was suppressed after I3C treatment as expected. We also observed a change in p53 localization: the expression of p53 in the cytoplasm and nucleus increased after I3C treatment. As for MDM2, the expression levels were decreased in the cytoplasm but increased in the nucleus, possibly because I3C indirectly inhibited the expression of phosphorylated MDM2 in the nucleus, reducing the ubiquitination of p53, thereby promoting the expression of p53 [42]. In addition, immunofluorescence colocalization results showed that the colocalization of p53 and MDM2 was decreased, therefore reducing the interaction of both (Figure 6). Meanwhile, the protein level of p53 was further increased after the combined treatment of I3C and MG-132. The inhibition of MDM2 expression by siRNA-p53 further confirmed that MDM2 is a downstream gene of p53, and I3C can affect the feedback inhibition between p53 and MDM2 (Figure 7). These results demonstrate that I3C stabilizes the activity of p53 by inhibiting its degradation by MDM2.

## 4. Materials and Methods

### 4.1. Cell Line, Reagents, and Treatment

The human liver cancer HepG2 cells were purchased from the Cell Bank of Type Culture Collection of the Chinese Academy of Sciences (Beijing, China) and cultured in DMEM (C11995500BT, Gibco, Waltham, MA, USA) containing 10% fetal bovine serum (FBS, 04-001-1A, BI, Herzliya, Israel) and 1% penicillin and streptomycin (P1400, Solarbio, Beijing, China) under 37 °C and humidified 5% CO_2_. Indole-3-carbinol (I3C), dissolved in dimethyl sulfoxide (DMSO, D5879, Sigma-Aldrich, St. Louis, MO, USA) was purchased from Sigma-Aldrich (I7256, St. Louis, MO, USA). Pifithrin-α (PFT-α, S1816) was purchased from Beyotime Biotechnology (Shanghai, China).

### 4.2. Cell Counting Kit-8 (CCK8) Assay

HepG2 cells were seeded into a 96-well plate at 5000 cells per well. After 24 h, the cells were treated with DMSO or different doses of I3C for 12, 24, and 48 h, respectively. After I3C treatment, 10 μL of CCK8 reagent (CA1210, Solarbio, Beijing, China) was added to each well and incubated for 1.5 h at 37 °C. The absorbance was measured at 450 nm by a microplate reader (Synergy H1, BioTek, Winooski, VT, USA). The values were recorded as mean ± SD, *n* = 6; the blank growth medium was used as the negative control. The cell survival percentage was calculated by a ratio of recorded value to the control.

### 4.3. Colony Formation Assay

HepG2 Cells (2000 cells per well) were seeded in 35 mm plates and incubated at 37 °C [43]. After overnight incubation, cells were treated with 200 μM I3C for 12 h. After replacing the medium, cells were incubated for 10 days. Plates were rinsed with phosphate-buffered saline (PBS, P1022, Solarbio, Beijing, China) and 2 mL of fixation reagent methanol was added for 15 min. Next, the cells were washed with PBS again and stained with 0.1% crystal violet (G1063, Solarbio, Beijing, China) for 10 min at room temperature. Plates were imaged using a digital camera (D7500, Nikon, Tokyo, Japan).

### 4.4. Wound Scratch Assay

Cell migration was measured by wound scratch assay. HepG2 (7 × 10^5^ cells/plate) cells were seeded into six-well plates and incubated in a normal growth medium. After 24 h, scratched wounds were gently created in the monolayer with a sterile 200 µL pipette tip across the center of the well. After removal of floating cells, cells were cultured with 1% serum medium and then treated with 200 μM I3C. Cell migration into the wound space was measured at 0, 24, and 48 h after treatment. Photographs were taken with the microscope (magnification, ×100; DS-Fi2, Nikon, Tokyo, Japan), and images were analyzed using the ImageJ software. Wound closure was determined according to the wound area.

### 4.5. Glucose Consumption and Lactate Release Assay

Cells were seeded into six-well plates at a density of 5 × 10^5^ cells in 2 mL DMEM per well and cultured overnight. Cells were then treated with or without 200 μM I3C for 12 h. Culture media were collected and centrifuged at 1000 rpm for 5 min. Glucose consumption and lactate release in the supernatant were detected using a commercially available glucose assay kit (BC2505, Solarbio, Beijing, China) and lactate assay kit (BC2235, Solarbio, Beijing, China), respectively, according to the manufacturer’s instruction with minor modifications. Glucose consumption was calculated by deducting the glucose level in the cultured medium from the glucose level of the fresh medium. Lactate release was calculated by plotting the standard curve using the standard solution with different dilution to calculate the content of lactate in the culture media. All data were normalized by the cell numbers.

### 4.6. Isolation of Total RNA and Real-Time PCR

Total RNA was extracted from HepG2 cells using the TRIzol reagent (DP424, TIANGEN, Beijing, China) following the manufacturer’s instructions. For miRNA analysis, reverse transcription was performed using the miRNA 1st Strand cDNA Synthesis Kit (by stem-loop) (MR101, Vazyme, Nanjing, China). For mRNA analysis, 2 µL of the total RNA was reverse transcribed using the HiScript II First Strand cDNA Synthesis kit (R223-01, Vazyme, Nanjing, China). The levels of mRNA or miRNA were determined by a real-time PCR system (Lightcycler 96, Roche, Basel, Switzerland) using qPCR SYBR Green Master mix (Q711-02, Vazyme, Nanjing, China), which were normalized by the internal control, β-actin, and U6, respectively. Each sample was analyzed in triplicate using the 2^−ΔΔCt^-based fold change method. The primer sequences are shown in Table 1 and were synthesized by Sangon (Shanghai, China).

### 4.7. Western Blot Analysis

The whole-cell lysate was extracted from cultured HepG2 cells using RIPA lysis buffer (P0013C, Solarbio, Beijing, China) containing PMSF (P0100, Solarbio, Beijing, China). The protein concentration of the lysate was detected by BCA assay (E112, Vazyme, Nanjing, China). Thirty micrograms of whole-cell lysate were separated by 10% sodium dodecyl sulfate-polyacrylamide gel electrophoresis (SDS-PAGE) and transferred onto Polyvinylidene Fluoride (PVDF) membranes. Antibodies against β-actin (AF7018, 1:1000), LDHA (DF6280; 1:1000), Lamin B1 (AF5161, 1:1000), and p53 (AF0879, 1:1000) were purchased from Affinity Biosciences (Changzhou, China). Antibodies against PKM2 (4053, 1:1000) were purchased from Cell Signaling Technology (USA). Antibodies against MDM2 (1:200) and HSP90 (sc-13119, 1:1000) were purchased from Santa Cruz (Dallas, TX, USA). The membranes were incubated with HRP conjugated antirabbit (AS014, 1:5000, Abclonal, Wuhan, China) or antimouse secondary antibodies (AS003, 1:5000, Abclonal, Wuhan, China). The blots were detected by Biokit Technology Maxilumin™-WB Pico Chemiluminescence Substrate reagent (WB001, Baizhi, Beijing, China) and exposed to ChemiDoc (Tanon -4600SF, Shanghai, China). The gray value analysis was carried out by GraphPad Prism.

### 4.8. Nuclear and Cytoplasmic Fractionation

The nuclear protein extraction kit (R0050, Solarbio, Beijing, China) was employed to isolate nuclear and cytoplasmic protein from HepG2 cells according to the manufacturer’s guidelines. Briefly, cells were resuspended in the cytoplasmic extract reagent, vortexed for 10 s, and placed on ice for 5 min, then centrifuged at 16,000× *g* for 5 min at 4 °C. The supernatant was collected as the cytoplasmic extract. The pellet was resuspended in the nuclear extract reagent, vortexed for 15 s, and placed on ice for 10 min, then centrifuged at 16,000× *g* for 10 min at 4 °C. The supernatant was collected as the nuclear extract.

### 4.9. Determination of the Levels of Mitochondrial Membrane Potential

The levels of the mitochondrial membrane potential (MMP) were monitored using the mitochondrial membrane potential assay kit with JC-1 (C2006, Beyotime, Shanghai, China). JC-1 aggregates (red fluorescence) in healthy mitochondria, whereas it exerts in monomers (green fluorescence) in unhealthy mitochondria. Therefore, the red/green ratio is used as a sensitive measure of changes in MMP. HepG2 cells (5 × 103/well) were seeded into a 96-well culture plate and incubated at 37 °C. After 24 h, the cells were treated with 200 μM I3C. After six more hours, cells were rinsed and added with the JC-1 working solution. Cells were then incubated at 37 °C for 20 min. After incubation, cells were rinsed twice and the level of MMP was determined by the ratio of the intensity of red fluorescence to green fluorescence (Synergy H1, BioTek, Gene Company Limited, Winooski, VT, USA).

### 4.10. Small Interfering RNA (siRNA) Transfection

Twenty thousand HepG2 cells were seeded in a 6-well tissue culture plate, and transfection was started when the cell density reached 60–80%. Preparation of solution A: 2 μL of p53 siRNA (sc-29435, Santa Cruz, Dallas, TX, USA) or a negative control (sc-37007, Santa Cruz, Dallas, TX, USA) was added into 100 μL of siRNA transfection medium (sc-36868, Santa Cruz, Dallas, TX, USA); solution B: 2 μL of transfection reagent (sc-29528, Santa Cruz, Dallas, TX, USA) was added into 100 μL of siRNA transfection medium. Solutions A and B were mixed gently and incubated for 45 min at room temperature. Cells were rinsed once using DMEM medium; for each transfection, 0.8 mL of siRNA transfection medium was added into the solution of the A/B mixture, mixed well, and added to the cells. After 6 h of incubation, 2 mL of medium was added into each well. Cells were cultured for an additional 24 h. All of the reagents were purchased from Santa Cruz Biotechnology (Santa Cruz, Dallas, TX, USA).

### 4.11. Immunofluorescence Staining

Seventy-five thousand HepG2 cells (per well) were seeded on glass coverslips pretreated with TC (YA0350, Solarbio, Beijing, China). The following day, cells were treated with DMSO and I3C as explained previously for 12 h. Next, cells were rinsed twice with PBS and fixed in 4% paraformaldehyde (P1110, Solarbio, Beijing, China) for 15 min at room temperature. Then, cell permeation was performed with 0.5% Triton X-100 (T8200, Solarbio, Beijing, China) for 20 min. After cells were blocked with 1% bovine serum albumin (BSA, A8020, Solarbio, Beijing, China) for 30 min, the cell climbing slices were incubated with anti-p53 (1:200) and anti-MDM2 (1:50) overnight at 4 °C. After washing with PBS, we added goat antirabbit lgG (H+L) Fluor488-conjugate (S0018, Affinity Biosciences, Changzhou, China) and antirabbit lgG (H+L) Fluor647-conjugate (S0014, Affinity Biosciences, Changzhou, China) for 1 h at room temperature. 4′,6-diamidino-2-phenylindole (DAPI, C0065, Solarbio, Beijing, China) was used for staining for 5 min at room temperature, and a 3 µL antifluorescence quenching solution was used to seal the slides. The mounted cells were analyzed using confocal microscopy (FV3000, OLYMPUS, Tokyo, Japan).

### 4.12. Identification of Candidate miRNAs

TargetScan (http://www.targetscan.org (accessed on 6 June 2021)) [44], miRcode (http://www.mircode.org/ (accessed on 6 June 2021)) [45], and starbase (http://starbase.sysu.edu.cn (accessed on 6 June 2021)) [46] databases were used to predict the target miRNAs of LDHA. RNAlocate (http://www.rna-society.org/rnalocate/index.html (accessed on 17 June 2021)) [47] was used to further validate the predictions.

### 4.13. Survival Analysis

The online database Kaplan–Meier Plotter (http://kmplot.com (accessed on 17 July 2021)) [48] was employed to evaluate the prognostic value of hepatocellular carcinoma samples of LDHA and miR-34a expression. Patient samples were categorized into high- and low-expression groups according to the following software’s auto setting of the best cutoff value. Logrank *p*-value < 0.05 was considered as statistically significant.

### 4.14. Construction of Protein–Chemical Interaction Networks by Bioinformatic Analysis

The interaction network between drugs and their targeted proteins was predicted using the STITCH database (http://stitch.embl.de/ (accessed on 8 July 2021)) [49]. The inputs for analysis were composed of I3C to predict molecular targets.

### 4.15. Statistical Analysis

For multigroup comparisons, one-way analysis of variance (ANOVA) was used in conjunction with the Newman–Keuls post-test. All of the experiments were repeated more than three times. *p* < 0.05 indicates a significant difference.

## 5. Conclusions

**Figure 8:**
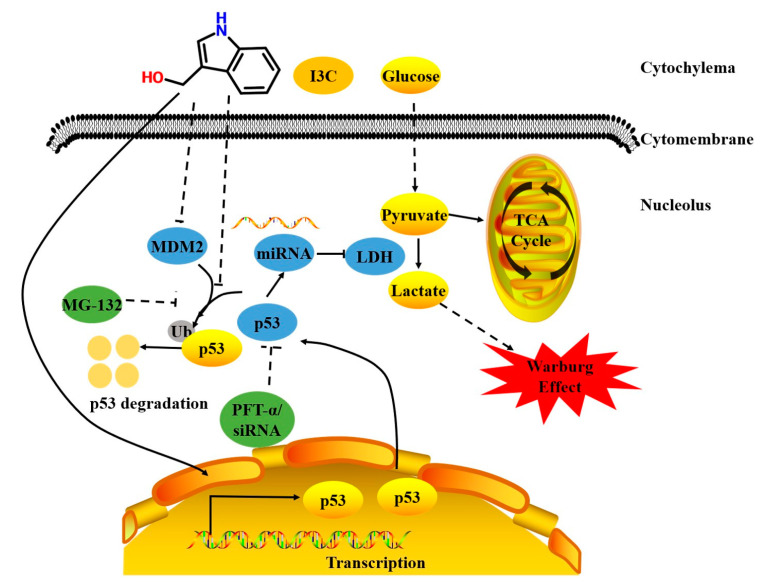
The schematic diagram of the action of I3C in liver cancer cells.

Taken together, we first demonstrated that I3C induces miR-34a, which then targets LDHA, a vital enzyme for aerobic glycolysis, by inhibiting the degradation of p53 by MDM2, thereby downregulating the expression of LDHA and suppressing aerobic glycolysis, leading to growth inhibition of liver cancer cells. Our study provides a theoretical and experimental basis for the plant source of I3C in the treatment of liver cancers (Figure 8). Our results are the first to demonstrate the therapeutic potential of I3C for human liver cancer. Subsequent studies will focus on the in-depth mechanisms of action and the pharmacal kinetics of I3C using mouse models of liver cancer.

## Figures and Tables

**Table 1:** The primer sequences for real-time PCR.

Gene Name	Sequences
IDH1	Forward: 5′-TCAGTGGCGGTTCTGTGGTA-3′
	Reverse: 5′-CTTGGTGACTTGGTCGTTGGT-3′
LDHA	Forward: 5′-ATGGCAACTCTAAAGGATCAGC-3′
	Reverse: 5′-CCAACCCCAACAACTGTAATCT-3′
PKM2	Forward: 5′-CTGTGGACTTGCCTGCTGTG-3′
	Reverse: 5′-TGCCTTGCGGATGAATGACG-3′
p53	Forward: 5′- ACAACGTTCTGTCCCCCTTG-3′
	Reverse: 5′- TCATCTGGACCTGGGTCTTC -3′
β-actin	Forward: 5′-CATGTACGTTGCTATCCAGGC-3′
	Reverse: 5′-CTCCTTAATGTCACGCACGAT-3′
miR-34a	Forward: 5′-TGGCAGTGTCTTAGCTGGTTGT-3′
	Reverse: 5′-GTCGTATCCAGTGCAGGGTCCGA GGTATTCGCACTGGATACGAC-3′
miR-449a	Forward: 5′-TGGCAGTGTATTGTTAGCTGGT-3′
	Reverse: 5′-GTCGTATCCAGTGCAGGGTCCGAGG TATTCGCACTGG ATACGAC-3′
U6	Forward: 5′-CTCGCTTCGGCAGCACA-3′
	Reverse: 5′-AACGCTTCACGAATTTGCGT-3′

## Data Availability

Data are contained within the article and Supplementary Materials.

## Supplementary Materials

The following supporting information can be downloaded at: https://www.mdpi.com/article/10.3390/ph15101257/s1, Figure S1: Cell viability was detected by CCK8 assay.
