# Supplementary material for: Indole-3-Carbinol Stabilizes p53 to Induce miR-34a, Which Targets LDHA to Block Aerobic Glycolysis in Liver Cancer Cells
Source: Pharmaceuticals (Basel). 2022 Oct 13;15(10):1257. doi: 10.3390/ph15101257 (PMC9606903; doi:10.3390/ph15101257)
Supplement: Supplementary file 1 [file pharmaceuticals-15-01257-s001.zip › pharmaceuticals-1929193-supplementary.pdf]

Supplementary Materials

Figure

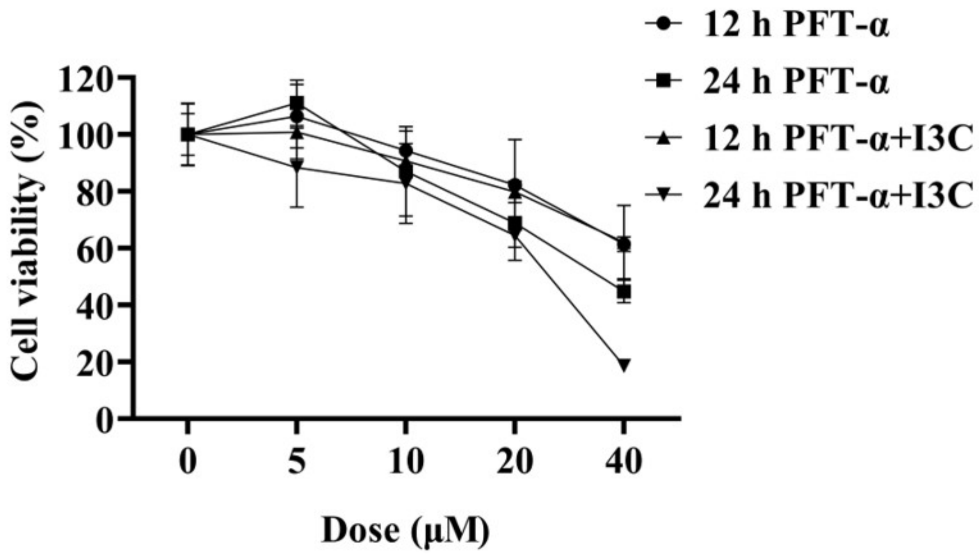

Figure S1. Cell viability was detected by CCK8 assay (n = 6). HepG2 cells were treated with different doses of PFT-α alone and combination with I3C for 12 and 24 h. Untreated cells were used as controls.
